# Long-term patterns of hillslope erosion by earthquake-induced landslides shape mountain landscapes

**DOI:** 10.1126/sciadv.aaz6446

**Published:** 2020-06-05

**Authors:** Jin Wang, Jamie D. Howarth, Erin L. McClymont, Alexander L. Densmore, Sean J. Fitzsimons, Thomas Croissant, Darren R. Gröcke, Martin D. West, Erin L. Harvey, Nicole V. Frith, Mark H. Garnett, Robert G. Hilton

**Affiliations:** 1 Department of Geography, Durham University, Durham, UK.; 2 Institute of Hazard, Risk and Resilience, Durham University, Durham, UK.; 3 State Key Laboratory of Loess and Quaternary Geology, Institute of Earth Environment, Chinese Academy of Sciences, Xi’an, China.; 4 CAS Centre for Excellence in Quaternary Science and Global Change, Xi’an, China.; 5 School of Geography, Environment and Earth Science, Victoria University of Wellington, Wellington, New Zealand.; 6 School of Geography, University of Otago, Dunedin, New Zealand.; 7 Department of Earth Sciences, Durham University, Durham, UK.; 8 NERC Radiocarbon Facility, Rankine Avenue, East Kilbride, Glasgow, UK.

## Abstract

Widespread triggering of landslides by large storms or earthquakes is a dominant mechanism of erosion in mountain landscapes. If landslides occur repeatedly in particular locations within a mountain range, then they will dominate the landscape evolution of that section and could leave a fingerprint in the topography. Here, we track erosion provenance using a novel combination of the isotopic and molecular composition of organic matter deposited in Lake Paringa, New Zealand. We find that the erosion provenance has shifted markedly after four large earthquakes over 1000 years. Postseismic periods eroded organic matter from a median elevation of 722 ^+329^/_−293_ m and supplied 43% of the sediment in the core, while interseismic periods sourced from lower elevations (459 ^+256^/_−226_ m). These results are the first demonstration that repeated large earthquakes can consistently focus erosion at high elevations, while interseismic periods appear less effective at modifying the highest parts of the topography.

## Teaser

Large earthquakes shape mountain landscapes at high elevations.

## INTRODUCTION

The steep topography of active mountain belts emerges from the interplay between tectonic uplift, river incision, and bedrock landsliding. When the competing processes of uplift and river incision steepen hillslopes to the point where they reach the threshold for gravitational failure, hillslope erosion by landsliding acts to limit relief in these landscapes (*1*–*3*). Thus, landsliding can be viewed as a passive response to changes in the rate of fluvial incision in traditional views of “threshold” conditions (*1*). However, both empirical and modeling studies have shown that landslides dominate hillslope erosion rates and provide sediment to river systems that, in turn, mediates fluvial incision rates (*4*–*6*). They may also drive drainage divide migration and river piracy that controls landscape evolution (*7*–*9*). Hence, the spatial and temporal distribution of landsliding should exert a first-order influence on landscape evolution in active mountain belts (*10*).

Storms and earthquakes can trigger thousands of landslides (*11*, *12*) with different spatial patterns because of the way they influence body forces within hillslopes and promote gravitational failure. For instance, storm rainfall causes landsliding by increasing pore fluid pressures, which can be exacerbated at lower elevations on hillslopes due to seepage (*13*, *14*). Thus, rainfall-induced landsliding is thought to erode lower elevations on hillslopes (*15*). In contrast, earthquakes may preferentially trigger landslides at high elevations on hillslopes (*16*, *17*), due to topographic amplification of strong ground motions at ridge crests (*18*, *19*). These observations underpin the hypothesis that hillslope morphology in mountain belts is controlled by the dominant landslide triggering process (*16*, *20*). This hypothesis is supported by differences in hillslope morphology between mountain belts dominated by either rainfall- or earthquake-induced landsliding (*20*). However, a definitive test requires demonstration that the spatial pattern of landsliding predicted by theory and observed during discrete trigger events translates into coherent spatial patterns of hillslope erosion over millennial time scales (*10*, *20*).

Constraining spatial patterns of hillslope erosion over multiple trigger events is problematic, however, because of the long recurrence times of those events. Remote sensing is the most effective way of observing spatial patterns of landsliding, but these datasets tend to span single events to a few decades at most (*2*, *5*, *12*). The short duration of these records implies that we cannot assess whether spatial patterns linked to landslide triggering events persist over the time scales that are relevant to landscape evolution. Sedimentary basins with catchments draining mountain topography have the potential to archive millennial-scale records of earthquake- and rainfall-driven landsliding and related sediment flux (*21*–*24*). If we were able to resolve the provenance of sediment in these records, particularly in terms of the elevation of erosion, these records could allow us to track longer-term spatial patterns of hillslope erosion by landsliding.

**Fig. 1:**
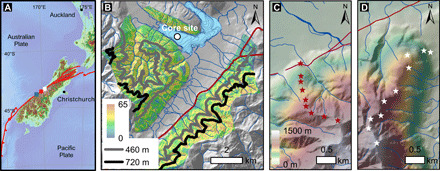
The study setting and topography of the Lake Paringa catchment and soil sample elevation transects. (**A**) Alpine Fault in southern New Zealand and the study locations. The blue, red, and white squares show the locations of (B), (C), and (D), respectively. (**B**) The location of Lake Paringa and the sediment core PA6m1. The colored polygon shows the slope of the source catchment of Lake Paringa. The gray and black lines are the 460- and 720-m contours, which are the median of inter- and postseismic sediment erosion elevation. (**C**) Locations of soil samples from the Mount Fox transect overlain on topography from a digital elevation model with 8-m resolution. (**D**) Locations of soil samples along the Alex Knob transect.

Here, we develop an approach to reconstruct the past elevation and depth of erosion using a novel combination of geochemical techniques applied to organic matter in lake sediments. Specifically, we analyze the stable carbon isotope ratio of bulk organic matter (δ^13^C_org_), nitrogen isotope ratios of the bulk sediment (δ^15^N), the abundance and ratios of *n*-alkanes (carbon preference index, CPI_*n*-alkanes_), the radiocarbon activity of bulk organic matter (fraction modern, *F*^14^C), and the hydrogen isotopic composition of long-chain *n*-alkane (δD_C29-*n*-alkane_) (see Materials and Methods). These variables are expected to vary with the elevation of plant growth or soil formation (*25*, *26*) and with soil depth (*27*, *28*). We combine these measurements to track erosion provenance in lake sediments fed by river catchments that drain the range front of the western Southern Alps, New Zealand (Fig. 1; Materials and Methods). We first assess the composition of soil organic matter collected across elevation and depth gradients, before investigating the sedimentary record from Lake Paringa, which archives cycles of earthquake- and rainfall-driven sediment flux over a thousand years (*23*). Our analysis reveals that earthquake-induced landsliding preferentially erodes high elevations in the catchment, shaping hillslope morphology and potentially driving drainage divide migration.

## RESULTS

### The geochemistry of organic matter in soils of the western Southern Alps

To assess the degree to which the geochemistry of organic matter in eroded sediments encodes the elevation of its source or the erosion depth, we first examine soils collected along two elevation transects on the western flank of the Southern Alps (Fig. 1 and fig. S1; Materials and Methods). Samples collected from the Mount Fox trail, located ~55 km northeast of Lake Paringa, cover elevations from 250 to 1160 m. Samples from the Alex Knob trail cover elevations from 290 to 1290 m and are located further to the northeast (Fig. 1 and fig. S1; see Materials and Methods). The Mount Fox transect was used to develop relationships between soil organic geochemistry and elevation due to its closer proximity to Lake Paringa and similar soil types (fig. S1, D and E), while the Alex Knob transect was used as an independent test of these relationships.

**Fig. 2:**
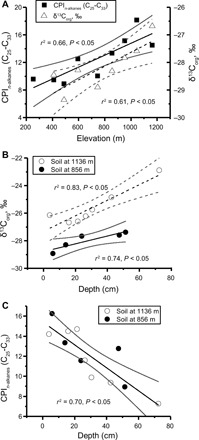
The relationship between δ^13^C_org_ and CPI_*n*-alkanes_ of organic matter in soils from Mount Fox as a function of elevation and soil depth. (**A**) Positive linear relationship between CPI_*n*-alkanes_ and δ^13^C_org_ of soil A horizons with the sampling elevation. (**B**) Positive linear relationship between δ^13^C_org_ and depth at two soil profiles. (**C**) Negative linear relationship between CPI_*n*-alkanes_ and depth at two soil profiles.

At Mount Fox, the δ^13^C_org_ values of soil A horizons are positively correlated with sample elevation (*r*^2^ = 0.61, *n* = 8, *P* < 0.05; Fig. 2A), varying by ~2.7‰ over an elevation range of 750 m (Fig. 2A). These trends are similar to those observed in other mountain forests for plants (*25*) and soil organic matter (*29*). The increase in δ^13^C_org_ values with elevation may reflect a combination of declining atmospheric pressure and *P*co_2_ (partial pressure of CO_2_) concentrations, which influences isotopic fractionation during photosynthesis (*25*, *30*), and/or partial pressure of oxygen (*P*o_2_) concentrations (*31*). δ^13^C_org_ is also positively correlated with the sampling depth of soil organic matter (Fig. 2B). An increase in δ^13^C_org_ values with increasing soil depth is consistent with the roles of organic matter degradation (*32*) and addition of partially weathered rock-derived organic carbon (OC) (*33*).

The concentrations of soil *n*-alkanes across depth profiles vary by three orders of magnitude with ranges from 1.4 to 1510.1 μg g^−1^ soil (Σalk) or 0.1 to 39.7 mg g^−1^ OC on an OC-normalized concentration basis (Λalk). The molecular abundance distributions of *n*-alkanes show a significant odd-to-even carbon number preference and higher abundances of C_27_, C_29_, and C_31_ *n*-alkanes (table S1). The CPI of the long-chain *n*-alkanes (see Materials and Methods) has an average value across all soil samples of 12.0 ± 2.9 [±SD (σ) unless otherwise stated, *n* = 19], similar to other terrestrial samples (*34*, *35*). We find that the CPI_*n*-alkanes_ values of the soil A horizons are positively correlated with the sample elevation (Fig. 2A). This is consistent with measurements from some other mountain soils (*36*) and with the observed links between soil temperature and CPI_*n*-alkanes_ values (*37*). This CPI elevation link may reflect changing rates of degradation of soil organic matter with elevation (and thus with temperature), or it could also reflect a change in vegetation type (*38*). We find that the CPI_*n*-alkanes_ values of two soil profiles show a negative correlation with soil depth (Fig. 2B), with decreasing CPI_*n*-alkanes_ again reflecting increased degradation with soil depth.

The δ^15^N and *F*^14^C values of soil organic matter vary with depth, but not elevation (fig. S2), consistent with these variables being most strongly linked to organic matter degradation through time as soils develop (*27*, *28*, *39*). The δD_C29-*n*-alkane_ values of soil A horizons are not correlated with elevation at Mount Fox, but they do plot within the broad negative trend between δD_C29-*n*-alkane_ and elevation defined by Zhuang *et al*. (*40*) in the Haast River and nearby catchment to the south of Lake Paringa (fig. S3).

In terms of tracking provenance, the paired δ^13^C_org_ and CPI values of organic matter offer a tool to constrain both the elevation and depth of eroded soil in this setting. This is because δ^13^C_org_ and CPI values are both positively correlated with elevation (Fig. 2A), while they are anticorrelated with soil depth (Fig. 2, B and C). The δ^15^N and *F*^14^C values offer independent constraint on the soil depth, while δD_C29-*n*-alkane_ could independently track elevation.

### Lake sediment record from the western Southern Alps, New Zealand

A 6-m sediment core collected from Lake Paringa records four *M*_w_ (moment magnitude) > 7.6 earthquakes as rapidly deposited layers formed by coseismic subaqueous mass wasting (*23*, *24*). Previous studies have described these deposits and their chronology in detail (*23*, *24*). In summary, sediments between these coseismic deposits represent deposition over multiple seismic cycles, each characterized by a phase of postseismic and interseismic deposition. The core chronology is based on plant macrofossil ^14^C activity (*23*, *41*), and the coseismic deposits have been linked to independent constraints on the timing of past earthquakes (*42*, *43*). Elevated sediment accumulation after each earthquake demonstrates that the postseismic sediment flux is five times that of interseismic periods for, on average, ~50 years after each earthquake (*24*).

Lake Paringa is fed by catchments which drain steep and densely vegetated hillslopes close to the Alpine Fault. The Windbag Creek basin (31 km^2^) drains the range front and has a distribution of slope angles similar to larger adjacent catchments (*21*) and vegetated slopes from 16 to 1420 m. The vegetation and soils are similar to those sampled at Mount Fox (fig. S1). Because the catchment length is relatively short, it is likely that the composition of the suspended load riverine organic matter is not altered greatly during fluvial transport (*44*, *45*), in comparison to the study of Feakins *et al*. (*46*), where the mountain rivers fed 10 to 100 km of lowland floodplain. Thus, the isotopic and molecular compositions of lake sediment organic matter are likely to be representative of the hillslope source signatures.

The Lake Paringa core has been previously analyzed for total OC (TOC) to nitrogen ratio (C/N), δ^13^C_org_, *F*^14^C in bulk organic matter, and CPI_*n*-alkanes_ to assess the erosion of OC across the four seismic cycles (*21*). Postseismic phases are characterized by ^13^C-enriched OC, with a mean δ^13^C_org_ = −27.2 ± 0.6‰ (*n* = 101), and have higher C/N values of 18.5 ± 6.9; the interseismic phases have mean δ^13^C_org_ = −28.6 ± 0.5‰ and C/N values of 11.4 ± 1.4 (*n* = 63). These data reveal enhanced accumulation rates of biospheric carbon after four Alpine Fault earthquakes. Frith *et al*. (*21*) suggested that the shifts in δ^13^C and C/N are likely to reflect erosion and delivery of soil-derived carbon eroded by deep-seated landslides, but noted their lack of constraint on the composition of soils in the catchment headwaters.

**Fig. 3:**
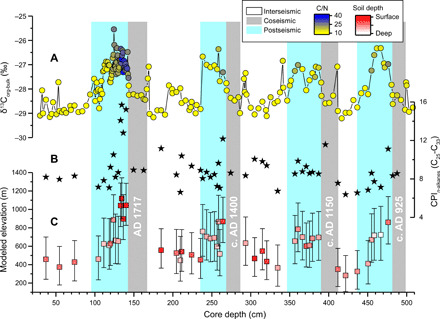
Geochemical analysis and modeled elevation and depth for core PA6m1. (**A**) Stable isotope composition of OC (δ^13^C_org_, ‰, analytical uncertainty smaller than the symbol size) from Frith *et al*. (*21*) and TOC to nitrogen ratio (C/N; colors). (**B**) Variation of CPI of long-chain *n*-alkanes. (**C**) Modeled elevation and depth (colors) of erosion from Eqs. 1 and 2. The gray bars show coseismic megaturbidites, a marker of large Alpine Fault earthquakes. The cyan bars show postseismic sediments [as per (*23*)].

**Fig. 4:**
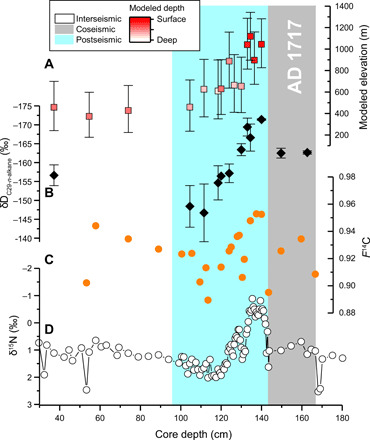
The evolution of predicted erosion provenance in Lake Paringa during the AD 1717 earthquake phase. (**A**) Predicted elevation and depth of organic matter as per Fig. 3, with gray bar showing the coseismic deposit, cyan showing the postseismic phase of deposition, and white showing the interseismic period [as per (*23*)]. (**B**) Hydrogen isotopic composition of long-chain *n*-alkanes (δD_C29-*n*-alkane_, ‰) (note the reverse scale). (**C**) Radiocarbon activity of bulk organic matter in the core (fraction modern, *F*^14^C). (**D**) Nitrogen isotopic composition of bulk organic matter.

To shed more light on the provenance of landslide-derived sediment deposited over multiple seismic cycles, in this study, we vastly increased the number of CPI_*n*-alkanes_ measurements (Fig. 3) from Frith *et al*. (*21*) and analyzed the δ^15^N of bulk organic matter and δD_C29-*n*-alkane_ (see Materials and Methods) down the core (Fig. 4). The mean δ^15^N values are 1.4 ± 0.5‰ in interseismic and 1.1 ± 0.9‰ in postseismic phases. These mean values hide the large changes in δ^15^N values following each earthquake, particularly after the AD 1717 event, which initially track δ^13^C_org_ values, before becoming decoupled after a period of deposition (fig. S4). The δ^15^N values are negatively correlated with the *F*^14^C of bulk organic matter in the core, with more ^14^C-enriched samples having lower δ^15^N values (Fig. 4 and fig. S5A). Decoupling of δ^13^C_org_, δ^15^N, and *F*^14^C values could be due to a shift in the provenance of erosion in terms of elevation (influencing δ^13^C_org_) and soil depth (influencing δ^13^C_org_, δ^15^N, and *F*^14^C).

The concentrations of *n*-alkanes (Λalk) do not vary between postseismic (mean Λalk = 1.1 ± 0.4 mg g^–1^ OC, *n* = 31) and interseismic (mean Λalk = 1.1 ± 0.3 mg g^–1^ OC, *n* = 16) deposits. However, the relative abundance of *n*-alkanes does vary. CPI_*n*-alkanes_ is slightly higher in postseismic (mean CPI_n-alkanes_ = 9.4 ± 2.3) than in interseismic (mean CPI_*n*-alkanes_ = 8.6 ± 1.4) deposits, although this is not statistically significant (*t* test, *P* = 0.07). Within postseismic phases, CPI_*n*-alkanes_ values are generally highest immediately after the earthquake marker (most notably for AD 1717 where we have the highest sampling resolution), before it gradually decreases to a relatively consistent interseismic value (Fig. 3).

The C_29_ *n*-alkane is thought to originate from higher terrestrial plants, and its hydrogen isotopic composition (δD) in river sediments has been shown to be sensitive to the elevation upstream of sample site (*26*, *40*). We have measured δD_C29-*n*-alkane_ for the last seismic cycle (AD 1717) and find that it is low immediately after the earthquake at −171.4 ± 0.2‰, before generally increasing through the postseismic period to −146.7 ± 7.6‰ (Fig. 4). On the basis of the broad pattern observed in soils from the Southern Alps, New Zealand (fig. S3), these patterns would suggest shifts in the source of C_29_ *n*-alkane deposited in the lake from high elevations immediately after the earthquake to lower elevations later in the postseismic phase and in the interseismic deposits.

### An empirical model of organic matter provenance

On the basis of the observed relationships between soil elevation (*Z*, m), soil depth (*H*, cm), δ^13^C_org_, and CPI_*n*-alkanes_ (Fig. 2), we proposed an empirical model to predict *Z* and *H* from paired δ^13^C_org_ and CPI_*n*-alkanes_ values in the lake core. Using only the Mount Fox data, we fit two planes to the discrete data (Materials and Methods)

$$\begin{array}{c} \delta^{13}C_{\text{org}}=3.9\pm 0.8\times 10^{-3}\cdot Z+4.3\pm 1.2\times 10^{-2}\cdot \\ H-31.9\pm 0.8(r^{2}=0.75,P<0.01) \end{array}$$ (1)

$$\begin{array}{c} {\text{CPI}}_{n-\text{alkanes}}=5.3\pm 2.1\times 10^{-3}\cdot Z-0.1\pm 0.03\cdot \\ H+9.8\pm 1.8\;(r^{2}=0.34,P<0.01) \end{array}$$ (2)

These models describe the first-order patterns in the data and provide a way to explore the lake record in terms of relative changes in *Z* and *H* over time. When δ^13^C_org_ and CPI_*n*-alkanes_ values are both high (or both low), organic matter in the samples is likely to derive mostly from surface soil horizons, and both variables track elevation (Fig. 2A). In contrast, discordance between these variables is modeled as a contribution from different soil depths (Fig. 2, B and C).

As a test of whether these models are supported for the wider study area, we first predict the δ^13^C_org_ values for the soil samples collected from the Alex Knob transect using Eq. 1 and the sample *Z* and *H* values, propagating the uncertainty on the empirical models (Materials and Methods). The predicted δ^13^C values are consistent with the measured values within uncertainty (*n* = 23, *r*^2^ = 0.32, *P* < 0.01; fig. S6), across a range of δ^13^C_org_ values from ~−30 to −25‰. This test of the model suggests that it can provide a realistic constraint on the elevation and depth of erosion from lake sediments. We note that CPI may not correlate with elevation in other settings [e.g., (*46*)], and so these empirical models may not be applicable outside this study area. We also note that our predicted depth derived from Eqs. 1 and 2 cannot track bedrock inputs due to its low OC content (*33*). The mixture of organic matter present in each lake sediment depth interval is assumed to represent the mean depth and mean elevation of the eroded materials.

**Fig. 5:**
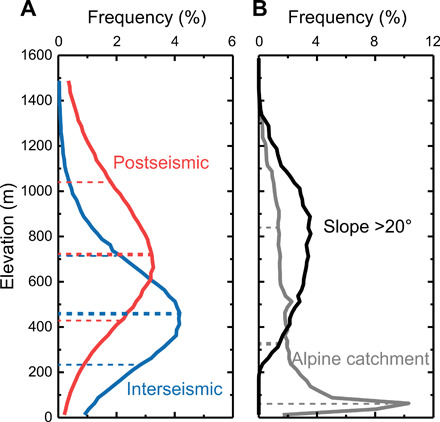
The elevation source of organic matter in Lake Paringa during the inter- and postseismic periods. (**A**) The blue and red lines show the elevation distributions of eroded soil for the whole record (Fig. 3C) during the inter- and postseismic period, respectively. The dashed lines show the 16th, 50th, and 84th percentiles of the distributions. (**B**) The gray line shows the elevation distribution of the alpine catchment (the west-flowing catchment of the Windbag River) with 16th, 50th, and 84th percentiles of the distribution as dashed lines. The black line is the distribution of slopes larger than 20° at alpine catchment. The frequency data of (A) and (B) have been binned in 25-m vertical intervals.

Using Eqs. 1 and 2, we predict the source elevation and soil profile depth of organic matter deposited in the lake sediment over four seismic cycles. The modeled elevation of erosion ranges from 283 (^+217^/_−176_) m to 1118 (^+225^/_−222_) m, while the depth ranges from 18 (^+18^/_−12_) cm to 63 (^+24^/_−20_) cm. The large uncertainties reflect the small size of the Mount Fox dataset and fit of the models (Fig. 2). We note that the median predicted erosion depth of the postseismic phases of deposition is 44 (^+24^/_−22_) cm and is within the large uncertainty band of the median of 36 (^+21^/_−18_) cm from the interseismic phases (Fig. 3). The median elevation of the postseismic phases of 722 (^+329^/_−293_) m, however, is substantially higher than the median interseismic of 459 (^+256^/_−226_) m (Fig. 5).

The predicted elevation is negatively correlated with the δD_C29-*n*-alkane_ measurements (*P* < 0.01, *n* = 10; fig. S5B) and fits with published measurements of lower δD_C29-*n*-alkane_ values in soils collected from higher elevations in the Southern Alps, New Zealand (*40*) and elsewhere (*26*, *36*, *46*). The predicted depth of erosion is positively correlated with δ^15^N values (*P* < 0.01, *n* = 30; fig. S5C) and is consistent with higher *F*^14^C values for lower depths of soil (*P* = 0.03, *n* = 5; fig. S5D).

## DISCUSSION

The δ^13^C_org_ and CPI_*n*-alkanes_ values of the sediment in Lake Paringa suggest enhanced delivery of material eroded from high elevations following large earthquakes (Fig. 5). For the last seismic cycle (after the AD 1717 event), the highest predicted median elevation of organic matter erosion occurs immediately after the earthquake, with values of 1041 (^+246^/_−227_) m and 1118 (^+225^/_−222_) m (Figs. 3 and 4). The source elevation of sediments then decreases toward the average predicted median elevation of the interseismic periods of 458 (^+242^/_−217_) m over a period of 78 ± 28 years (Figs. 3 and 4) (*23*).

While the empirical models that we use to quantify the elevation of eroded organic matter have considerable uncertainty (of ~200 to 300 m), the inference of post-earthquake mobilization of sediment from high elevations following the AD 1717 earthquake using δ^13^C_org_ and CPI_*n*-alkanes_ values is supported by the change in δD_C29-*n*-alkane_ values. The δD_C29-*n*-alkane_ values are correlated with the predicted elevation of erosion (fig. S5B). While the δD_C29-*n*-alkane_ soil data from this study and published data from the Southern Alps (*40*) are scattered, they do show an elevation gradient expected by the changing isotopic composition of precipitation (fig. S3), which is the foundation of using δD_C29-*n*-alkane_ as a proxy of paleoaltimetry (*40*).

The other three Alpine Fault earthquake cycles follow a similar pattern to that of the AD 1717 event, with higher predicted elevations of erosion after each earthquake (Fig. 3). For the c. AD 925 earthquake (the deepest in the core), it is clear that the modeled elevation is high after the earthquake and then generally decreases during the postseismic phase. The other two seismic cycles have postseismic phases that exhibit more complicated patterns of modeled elevation following the earthquakes, although elevation values generally remain higher than the mean values of the interseismic samples.

Unlike elevation, we find no significant difference in the predicted erosion depth of organic matter between the post- and interseismic phases of deposition. However, the mixture of surficial and deeper soil organic matter sources in both postseismic and interseismic phases supports landsliding as the primary mechanism of erosion on hillslopes in the catchment. Our observations are consistent with landsliding being the dominant process eroding hillslopes on the western range front of the Southern Alps in interseismic periods (*5*). There is also likely to be sorting of the eroded organic matter following a landslide triggering event. There is a suggestion in the post-AD 1717 event that the first organic matter to reach the lake is from high-altitude surface soils (Figs. 3 and 4), before deeper soils from those elevations reach the lake. In the future, a multi-geochemical parameter approach used here may help shed light on these important details of erosion and fluvial transport after widespread landsliding (*47*, *48*).

Our observations provide a test of the hypothesis that landslide trigger mechanisms influence the long-term spatial pattern of erosion on hillslopes in mountain belts (*20*). Because of increased pore fluid pressures low on hillslopes, rainfall-induced landsliding tends to erode low elevations due to seepage (*13*). Conversely, topographic amplification of ground motions at ridge crests and slope breaks during earthquakes results in earthquake-induced landsliding sampling high elevations (Figs. 3 and 5) (*18*, *19*).

These patterns manifest as large shifts in the provenance of sediment production that are recorded in the lake stratigraphy. The Lake Paringa record indicates that 43% of sediment flux from the catchment occurs from an average elevation of 722 (^+329^/_−293_) m during postseismic phases compared to 57% of the sediment flux from 459 (^+256^/_−226_) m during interseismic phases (Figs. 3 and 5) (*21*). It appears that over a thousand years in the catchments that feed Lake Paringa, storms and earthquakes combine to drive a relatively even distribution of hillslope erosion by landsliding in terms of elevation. Viewed in terms of a two-dimensional conceptual model (*20*), an impact of this could be the promotion of a planar hillslope morphology.

The Lake Paringa record is also relevant for our understanding of the rates and processes of drainage divide migration, and hence the dynamics of landscape evolution in mountain belts. The greater local relief as well as steeper channel profiles and hillslopes of west-flowing catchments on the Southern Alps range front show that they are “aggressors”, potentially capturing drainage area from their east-flowing counterpart with which they share a drainage divide (fig. S7) (*8*, *49*, *50*). On the basis of single triggering event mapping, landslides have been suggested to be the dominant process by which drainage divide migration occurs in mountain belts (*7*). Our results suggest that over the long term, earthquakes can be the dominant process driving landsliding at drainage divide elevations (Fig. 5 and fig. S7). For these reasons, we hypothesize that, in active mountain belts, large earthquakes are the primary process driving drainage divide migration. A corollary of this hypothesis is that the frequency of large earthquakes provides a direct link between tectonics, drainage divide migration, and the dynamics of landscape evolution. Our work demonstrates that extreme events, such as earthquakes and storms, may exert a first-order influence on landscape evolution through the coherent spatial patterns of erosion by landsliding that they generate.

## MATERIALS AND METHODS

### Study site and sediment core

The Southern Alps are formed by oblique convergence between the Australian and Pacific plates of 39.7 mm year^−1^ on a bearing of 245° (*51*), up to 80% of which is accommodated on the range-bounding Alpine Fault (*52*). The Alpine Fault is thought to rupture in major earthquakes (*M*_w_ > 7) with a quasi-periodic return period of 263 ± 68 years (*43*, *53*, *54*). The landscape of the western Southern Alps is dominated by steep slopes developed in metasedimentary bedrock and sufficient to support high rates of landsliding (*5*, *55*). Moisture derives predominantly from the Tasman Sea (*56*) and is transported by northwest winds, which drive precipitation of up to 10 to 12 m year^−1^ (*57*). The climate and tectonic setting drive erosion rates of up to 10 mm year^−1^ (*58*, *59*).

Lake Paringa is located ~3 km west of the Alpine Fault. The catchment draining to Lake Paringa has an area of ~60 km^2^ in the frontal western Southern Alps, with elevations that range from 16 to 1420 m (Fig. 1). Bedrock lithologies include mylonites and pelitic and psammitic schists of the Torlesse Terrane east of the Alpine Fault in the Matataketake Range, while Greenland Group quartzose metasandstones and mudstones of the Buller Terrane occupy the Collie Creek catchment and the hillslopes bordering the lake itself (*60*). The area is covered by temperate rainforest below ~800 m. Shrubs, grassland, and alpine herbs persist above the regional snowline at ~1250 m (fig. S1).

To constrain the composition of organic matter in soils as a function of elevation and soil depth, 19 soil samples were collected from different soil horizons across the elevation transect at Mount Fox, ~55 km northeast of the study area along the strike of the Alpine Fault. These soils represent the O (surface to 0.05 ± 0.04 m), A (0.05 ± 0.04 to 0.18 ± 0.06 m), E (0.18 ± 0.06 to 0.42 ± 0.10 m), B (0.42 ± 0.10 to 0.65 ± 0.09 m), and C (>0.65 ± 0.09 m) soil horizons. Samples were collected using a soil auger, with the depth of sample noted, and the soil logged in the field according to the World Reference Base for Soil Resources (WRB) classification system (e.g., O, A, E, B, and C layers).

A 6-m sediment core was collected from the center of Lake Paringa using a Mackereth corer (PA6m1). The core was correlated to a well-dated master core based on the radiocarbon analysis of 22 terrestrial macrofossils (*23*). It was recently used to assess the impact of large earthquakes on OC erosion (*21*). Four large earthquakes of magnitude *M*_w_ > 7.6 were recorded in the core at AD 1717, c. AD 1400, c. AD 1150, and c. AD 925 (*24*) and have been identified by three distinct sedimentary units: (i) coseismic megaturbidites, (ii) postseismic hyperpycnite stacks, and (iii) interseismic layered silts (*21*, *23*, *24*).

### Geochemical analyses

A total of 189 sediment samples were collected from core PA6m1 at 0.2- to 5.8-cm resolution by Frith *et al*. (*21*), in which OC concentration, [TOC] (%), the stable carbon isotopic composition of bulk organic matter, δ^13^C_org_ (‰), the radiocarbon activity of bulk organic matter (reported as fraction modern, *F*^14^C), and the total nitrogen concentration, [TN] (%), were analyzed. These samples were also analyzed for bulk nitrogen isotopic composition δ^15^N (‰). The detailed [TOC], δ^13^C_org_, and [TN] analytical methods can be found in the study of Frith *et al*. (*21*). In summary, 0.4 to 0.6 g of sample were ground to a powder and reacted with 20 ml of 0.25 M hydrochloric acid for 4 hours at approximately 70°C to remove any inorganic carbonate. In our study, our soil samples were processed using the within-capsule method. Approximately 2 mg of ground soil was added to a silver capsule (combusted within 2 weeks of use) and reacted with 1 M hydrochloric acid within the capsule. The capsule was then dried at 60°C in the oven, and the process was repeated twice more. For all samples, [TOC] and δ^13^C_org_ were determined by combustion at 1020°C in O_2_ within a Costech CHN elemental analyzer coupled via ConFlo III to a Thermo Delta V isotope ratio mass spectrometer (EA-IRMS) in the Stable Isotope Biogeochemistry Laboratory at Durham University. Total nitrogen content and stable nitrogen isotopic ratio (δ^15^N) were measured by combustion of untreated samples in an EA-IRMS with a Carbosorb trap to inhibit large CO_2_ peaks from affecting measurements. δ^13^C_org_ and δ^15^N values were normalized on the basis of measured values of several standards and reported relative to Vienna Pee Dee Belemnite (VPDB) and relative to air. Duplicates of the samples (*n* = 20) returned mean ± 1σ of [TOC] = ± 0.09%, δ^13^C_org_ = ± 0.08‰ and [TN] = ± 0.01% (*21*), and δ^15^N = ± 0.14‰. Radiocarbon measurements were made by accelerator mass spectrometry (AMS) as described by Frith *et al*. (*21*).

A subset of samples (*n* = 73) from periods of interest were selected across the lake sediment core and soil samples for the analysis of *n*-alkane abundance. Fifteen of these values were reported by Frith *et al*. (*21*) to indicate the predominantly terrestrial source of sediment. A total of 19 soil samples were also analyzed, including nine soil A horizons from across different elevations and two depth profiles. A detailed description of the *n*-alkane analysis can be found in the study of Frith *et al*. (*21*). In summary, total lipids were extracted in a microwave accelerated reaction system (MARS, CEM Corporation) in 12 ml of dichloromethane and methanol (3:1) before adding an internal standard (hexatriacontane; Sigma-Aldrich). The lipid extract was first saponified with 8% KOH in methanol/water (99:1) at 70°C for 1 hour. The “base” fractions were liquid-liquid extracted in 2.5 ml of pure hexane three times. The *n*-alkanes were separated by silica column chromatography, eluting with 4 ml of hexane. The abundance of *n*-alkanes was quantified using a gas chromatograph fitted with a flame ionization detector (Thermo Scientific TRACE 1310).

We report the concentration of individual homologs and the sum of the C_21_-C_35_ *n*-alkanes on a μg g^−1^ sediment/soil (Σalk) and μg g^−1^ OC basis (Λalk). The long-chain (C_25_-C_33_) CPI was calculated as

$$\begin{array}{c} \text{CPI}=1/2(\sum (C_{25}+C_{27}+\ldots C_{33})/\sum (C_{24}+C_{26}+\ldots C_{32}))+\\ 1/2(\sum (C_{25}+C_{27}+\ldots C_{33})/\sum (C_{26}+C_{28}+\ldots C_{34})) \end{array}$$ (3)

The hydrogen isotopic compositions (δD) of individual compounds were measured on 12 sediment and 7 soil samples using a Thermo GC-Py-IRMS system at the Department of Geography, Durham University. The system consists of a Trace 1310 GC coupled to a Thermo Delta V Plus via GC IsoLink II and a Thermo TG-5MS 30 m × 0.25 μm × 0.25 μm column. The alumina pyrolysis reactor was operated at 1420°C and conditioned with a CH_4_ backflush before use. H_2_ reference gas pulses were introduced at the start and end of each chromatogram to provide an isotope ratio reference point and to check the system stability during the run. Individual *n*-alkane isotope ratio values were corrected using a multipoint linear normalization of a C_16_-C_30_ *n*-alkane reference material (A6 standard provided by A. Schimmelmann, Indiana University, Bloomington). Reference *n*-alkanes from C_18_-C_30_ were used to generate the normalization curve, covering δD values from −29.7 to −263.0‰. The H_3_^+^ factor was determined on a daily basis with repeated measurements of H_2_ reference gas at varying dilutions at the start of each sequence. The mean H_3_^+^ factor was 2.719 ± 0.048 parts per million (ppm) mV^−1^ (±1σ, *n* = 17) over the 3-month analysis period, with day-to-day SDs of between 0.01 and 0.03 ppm mV^−1^. Reference materials A6 and B4 (provided by A. Schimmelmann, Indiana University, Bloomington) were used to check the validity of the H_3_^+^ factor calibration (using peak-based measurements) and to determine the minimum usable amplifier signal, which minimized the residuals, and gave an *r*^2^ value of at least 0.995 for the normalization plot. The concentration of the A6 *n*-alkane standard used for the linear normalization was adjusted to obtain amplifier intensities within this range (1000 to 4000 mV). Each sample was diluted and prerun to determine the optimum solvent volume required to fit within the amplifier signal range of the standards. One sample (PA6m1_s1_111.5) was found to be below the analytical range (700 mV) but has been included along with the uncertainty in Fig. 4.

The δD of the C_29_ *n*-alkane is reported here, as it is most abundant in most of the samples. δD values are reported relative to Vienna Standard Mean Ocean Water (VSMOW) and are expressed in per mil (‰). The precision (±1σ) of isotopic measurements of the standard is ±2‰ (*n* = 6) for C_29_ *n*-alkane. Each sample has been run twice, and the SD was reported as the analytical error. The chromatographic resolution was generally good for most of the *n*-alkanes with no coelution evident for the reported C_29_ *n*-alkane peak (fig. S8).

### Empirical model of organic matter provenance

Multiple linear regression was used to fit both δ^13^C_org_ and CPI to the elevation (*Z*, m) and depth (*H*, cm) for the soil samples from Mount Fox

$$\delta^{13}C_{\text{org}}=a_{1}\times Z+b_{1}\times H+c_{1}$$ (4)

$$\text{CPI}=a_{2}\times Z+b_{2}\times H+c_{2}$$ (5)

Parameters and their SEs were returned from the regression. The *Z* and *H* values can be determined by solving the equations for the lake sediment to reconstruct the elevation and depth of erosion.

The model is based on discrete soil sample values. In reality, erosion will integrate across a range of depths and elevations. To include this in the empirical model would require more detailed information on the spatial distribution of organic matter and biomolecules in the landscape than we currently hold. We therefore assume that erosion of a soil will mix materials in a linear manner, and that the resultant composition of sediments produced reflects the mean value of that mixture. In other words, the discrete values of *Z* and *H* returned for each lake sediment depth interval are assumed to be the mean value of a distribution.

A Monte Carlo simulation was used to take account of the uncertainty on the parameters. For each group of parameters, the elevation and depth calculations were repeated 10,000 times with random sampling of normally distributed scaling parameters. The elevation and depth values were reported on the basis of the median of the Monte Carlo distribution with lower and upper bounds defined by the 16th and 84th percentiles of the distribution, respectively.

## Supplementary Material

aaz6446_SM.pdf

## SUPPLEMENTARY MATERIALS

Supplementary material for this article is available at http://advances.sciencemag.org/cgi/content/full/6/23/eaaz6446/DC1
