# Supplementary material for: Long-term patterns of hillslope erosion by earthquake-induced landslides shape mountain landscapes
Source: Sci Adv. 2020 Jun 5;6(23):eaaz6446. doi: 10.1126/sciadv.aaz6446 (PMC7274776; doi:10.1126/sciadv.aaz6446)
Supplement: aaz6446_SM.pdf [file aaz6446_SM.pdf]

Science Advances
AAAS

advances.sciencemag.org/cgi/content/full/6/23/eaaz6446/DC1

Supplementary Materials for

Long-term patterns of hillslope erosion by earthquake-induced landslides shape mountain landscapes

Jin Wang*, Jamie D. Howarth, Erin L. McClymont, Alexander L. Densmore, Sean J. Fitzsimons, Thomas Croissant, Darren R. Gröcke, Martin D. West, Erin L. Harvey, Nicole V. Frith, Mark H. Garnett, Robert G. Hilton

*Corresponding author. Email: wangjin09@ieecas.cn

Published 5 June 2020, *Sci. Adv.* **6**, eaaz6446 (2020)
DOI: 10.1126/sciadv.aaz6446

This PDF file includes:

Figs. S1 to S8
Tables S1 and S2

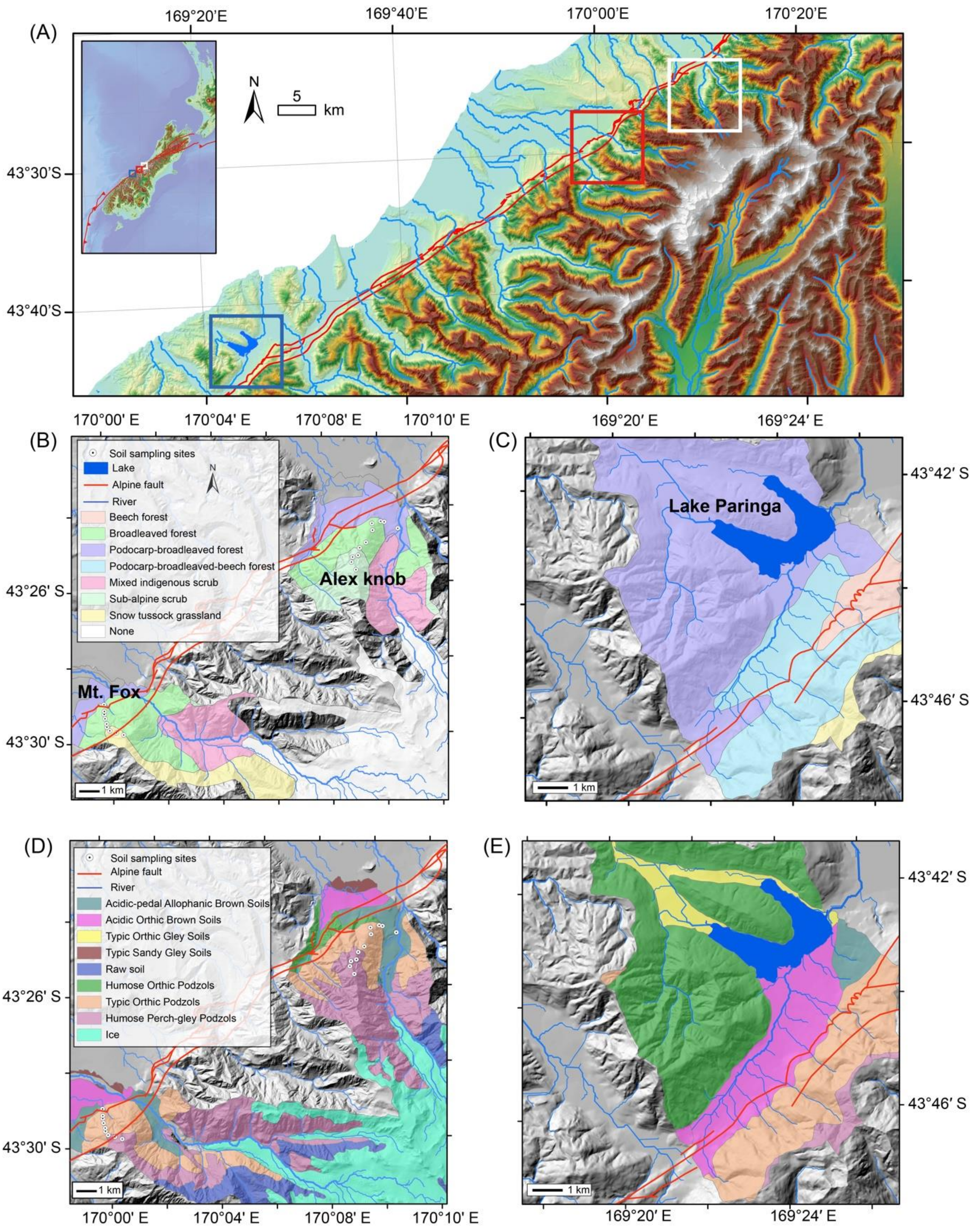

Fig. S1. The vegetation and soil types of the study location in southern New Zealand. (A) The study setting and topography of the Lake Paringa catchment. The blue, red and white rectangles show the locations of panels Fig. 1B, C and D. (B) The vegetation type of Mount Fox and Alex Knob track. The circles show the soil sampling locations. (C) The vegetation

type of Lake Paringa catchment. (D) The soil type of Mount Fox and Alex Knob track. (E) The soil type of Lake Paringa catchment. The vegetation and soil type data are from the New Zealand Land Resource Inventory (NZLRI; https://lris.scinfo.org.nz/).

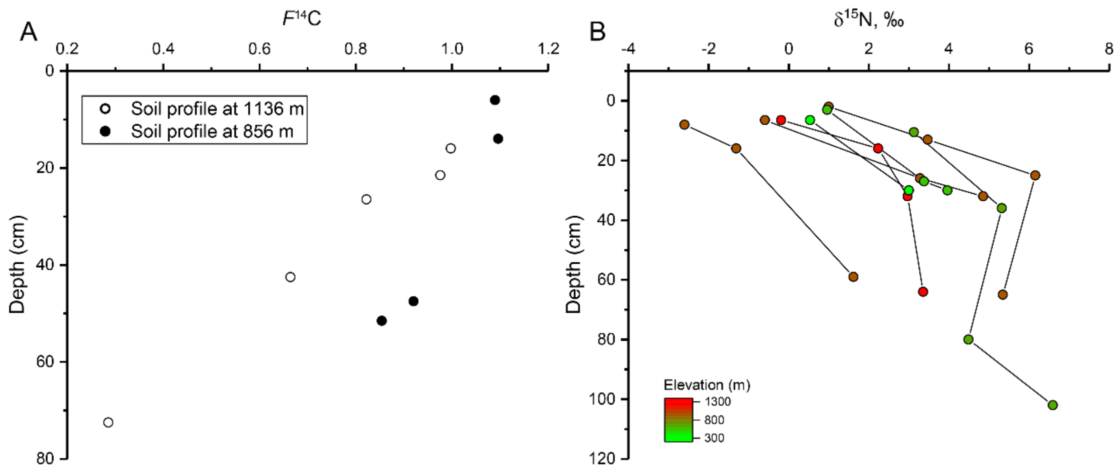

Fig. S2. The radiocarbon activity and stable nitrogen isotopic composition of the soil profile. (A) The filled and open circles show the $F^{14}C$ of organic carbon of soils collected at different depth at elevation 856 m and 1136 m of Mount Fox. (B) The circles show the $\delta^{15}N$ of soil collected at different elevation and depth at Alex Knob track.

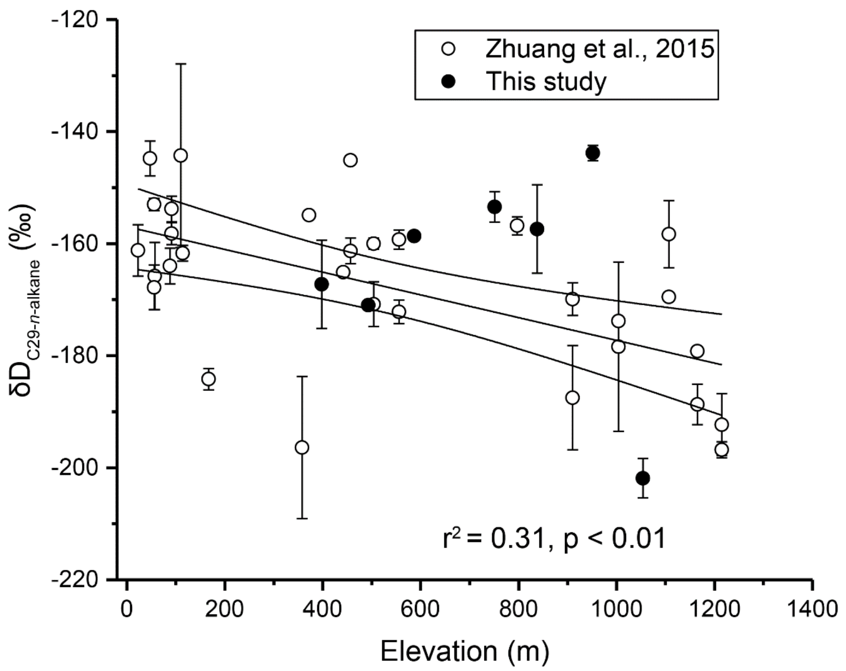

Fig. S3. Hydrogen isotope composition of C_{29} *n*-alkane ($\delta D_{C29\text{-}n\text{-alkane}}$) for Alpine soil samples. The open and filled circles are $\delta D_{C29\text{-}n\text{-alkane}}$ of soil collected in the Haast River and nearby catchment from Zhuang et al. (*40*) and Mount Fox of this study, respectively. The line is the linear regression for soils of Zhuang et al. (*40*), shown with 95% confidence intervals. Whiskers are the standard deviation of duplicated measurements.

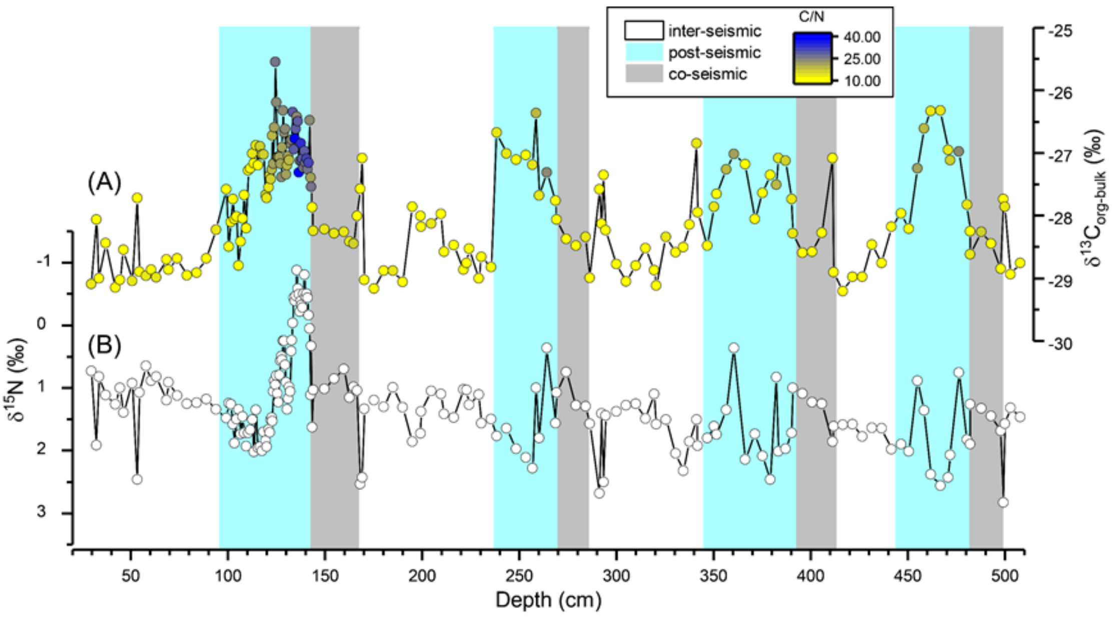

Fig. S4. Comparison of organic carbon and nitrogen stable isotopic ratios throughout the core PA6m1. (A) the stable isotope composition of organic carbon ($\delta^{13}C_{org}$) and the total organic carbon to nitrogen ratio. (B) the bulk stable nitrogen composition ($\delta^{15}N$). The grey bar shows earthquake marker, cyan showing the post-seismic phase of deposition, and white showing the inter-seismic period as per Fig. 3.

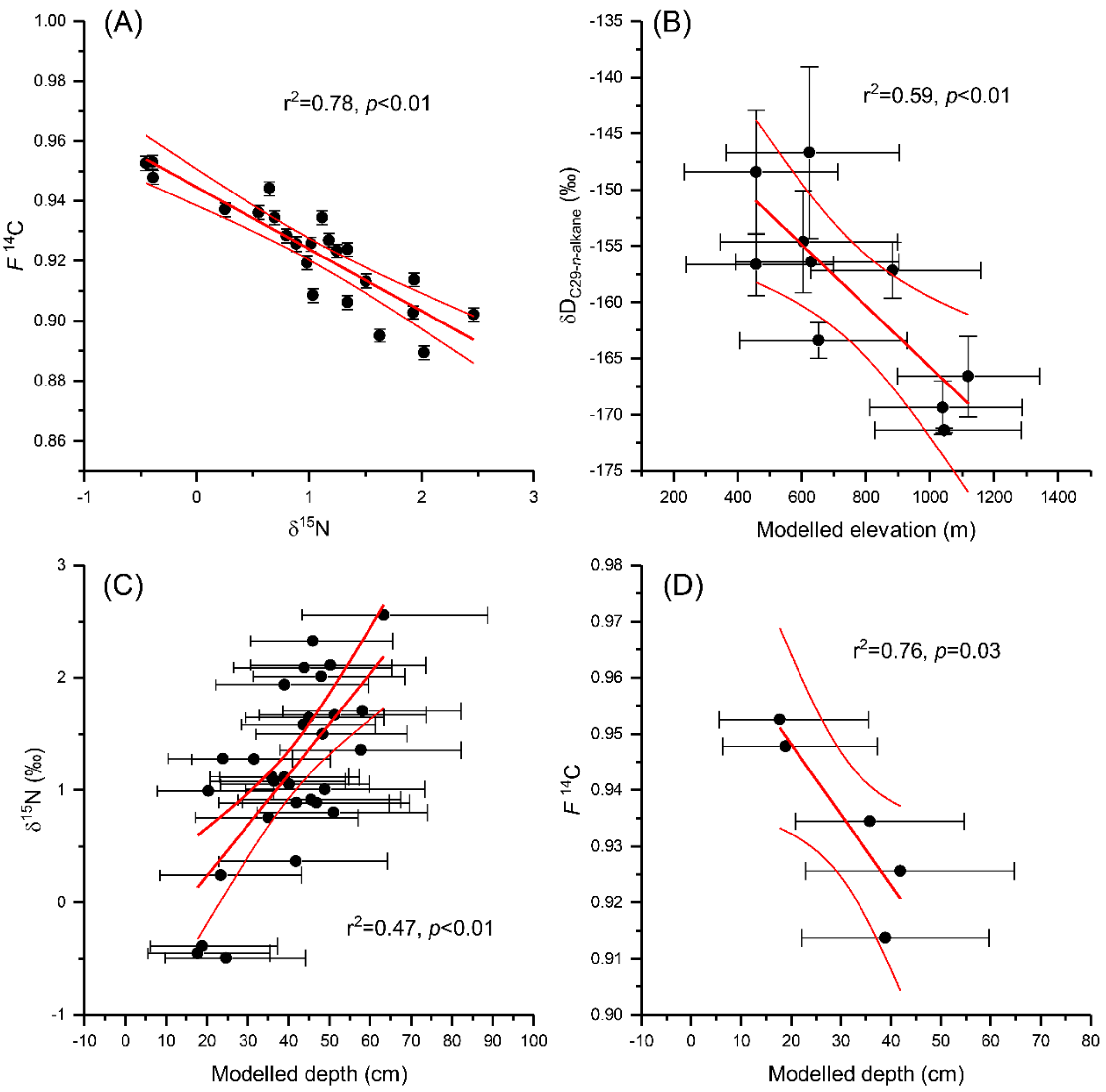

Fig. S5. Provenance predicted from $\delta^{13}C_{org}$ and $CPI_{n\text{-alkanes}}$ with equations 1 and 2 for the Lake Paringa sediment core compared to independent geochemical proxies. (A) The relationship between the Bulk $F^{14}C$ and $\delta^{15}N$. (B) Predicted elevation and $\delta D_{C29\text{-}n\text{-alkane}}$ values in the core. (C) $\delta^{15}N$ and predicted soil depth. D) $F^{14}C$ and predicted soil depth. Whiskers are analytical uncertainty and uncertainty propagated through the empirical model.

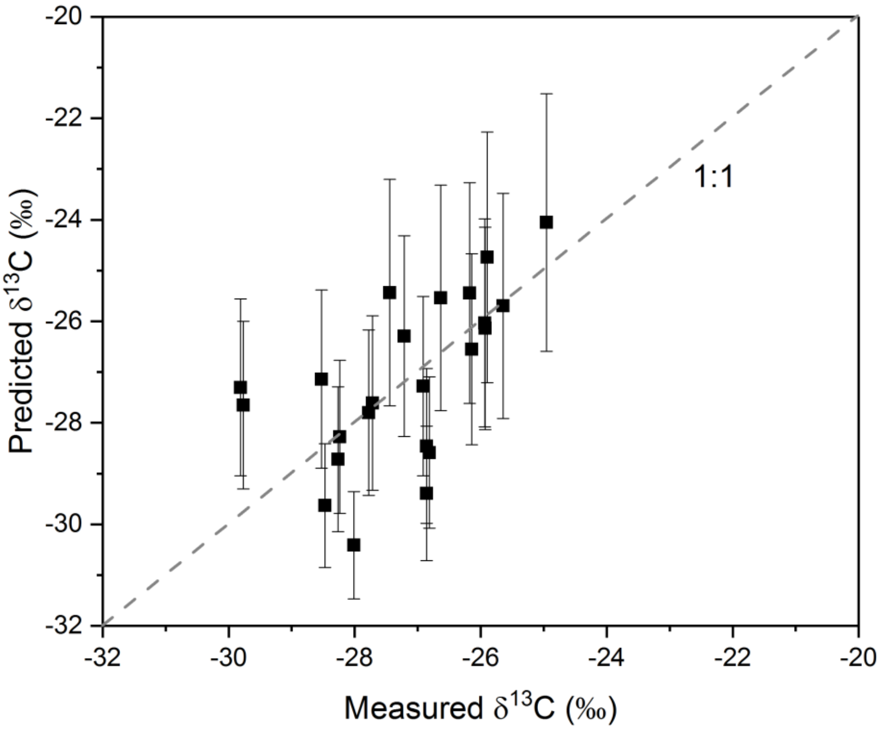

Fig. S6. Comparison between measured $\delta^{13}C_{org}$ values in soils from Alex Knob track (Fig. 1) versus those predicted based on Equation 1. Dashed line is 1:1.

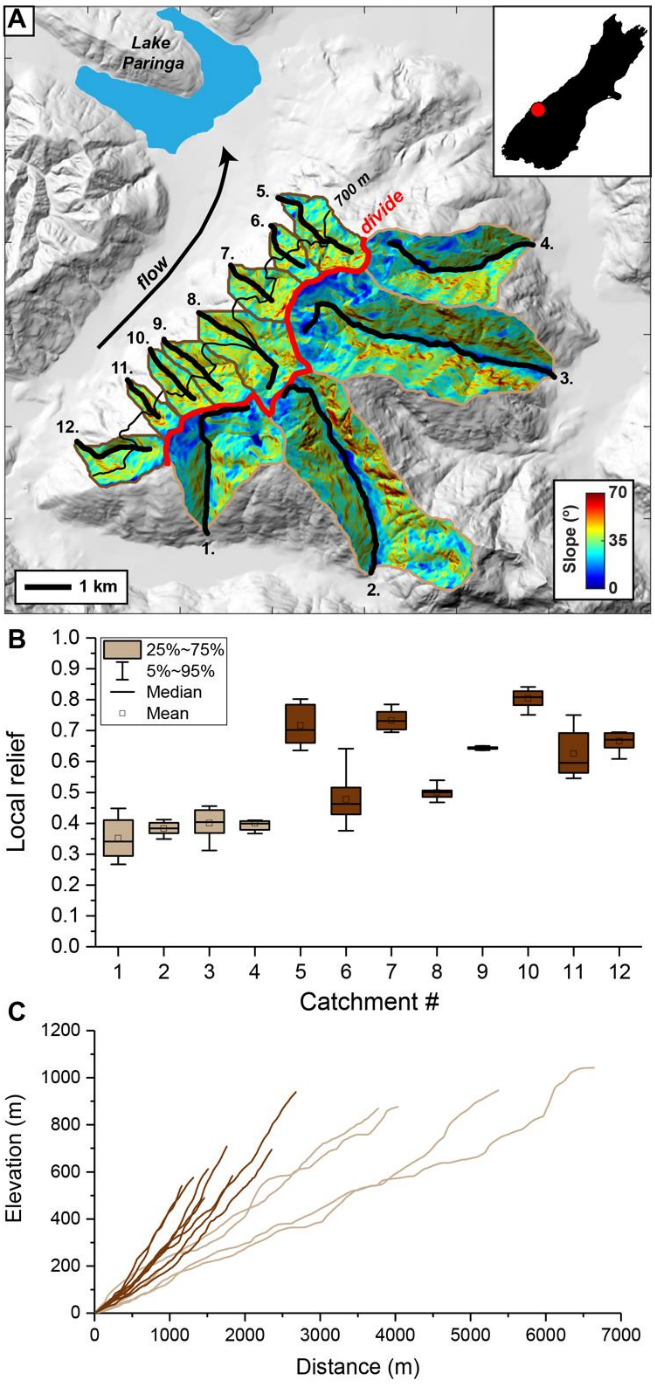

Fig. S7: Topographic metrics for the Southern Alps range front that drains into the Windbag basin of Lake Paringa. (A) Hillslope angle of catchments and drainage divide of west and east flowing drainages. (B) Local relief and (C) gradient for the east and west draining catchments. Higher hillslope relief and local relief in west draining catchments support eastward divide migration as west draining catchments capture the headwaters of their east facing counterparts.

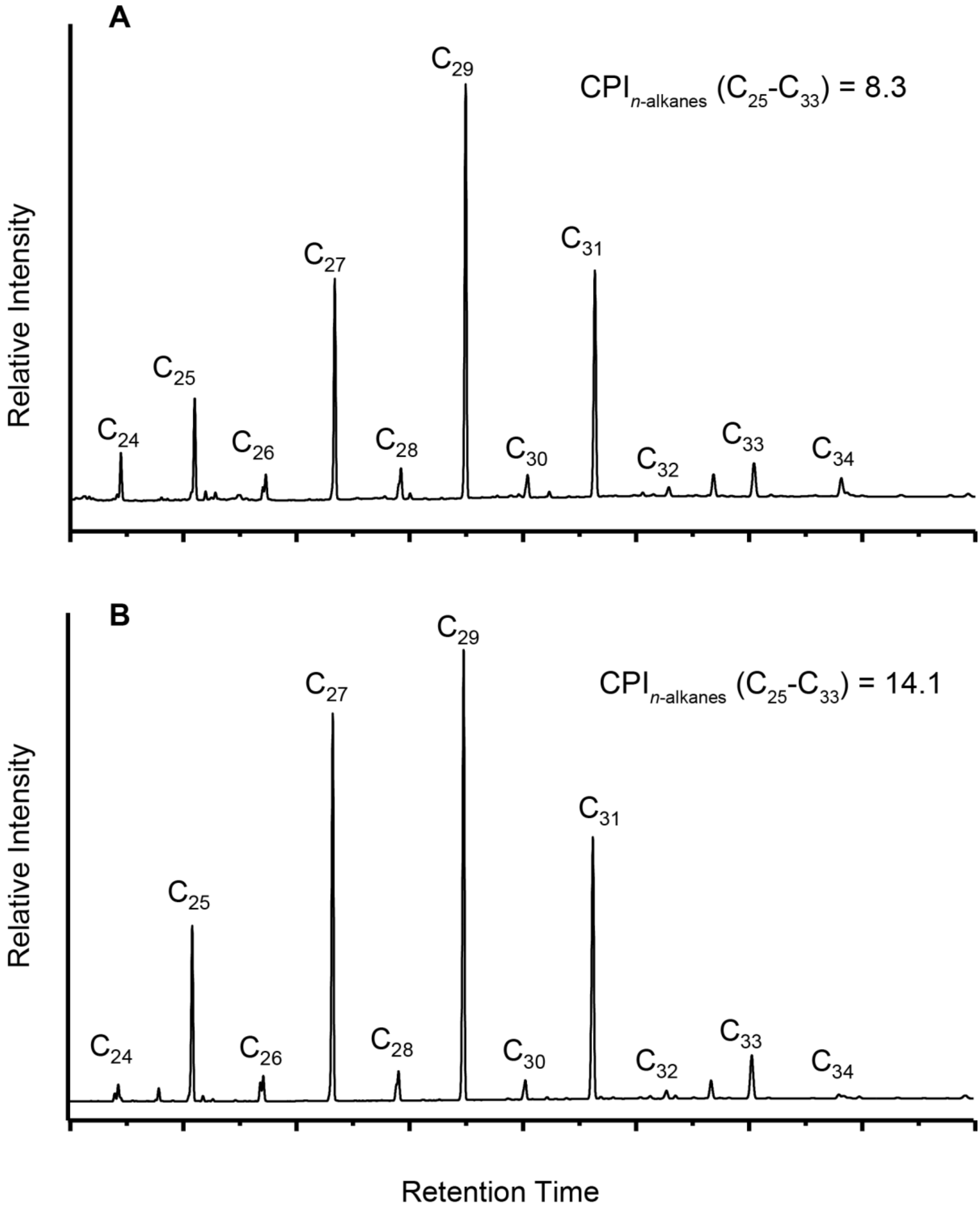

Fig. S8: GC/FID chromatogram of the sediment samples of Lake Paringa. Numbers on peaks are the carbon numbers of *n*-alkanes.

Table S1. *n*-alkanes abundance of soil and sediment samples

ID	*n*-Alkanes (ug/g sediment)															OC (%)	$\delta^{13}C$ (‰)	Σalk (ug/g sediment)	Λalk (mg/g OC)	$CPI_{n\text{-alkanes}\text{-}C_{25}\text{-}C_{33}}$	Elevation (m)	Depth (cm)	$F^{14}C$	± 1 σ	Publication Code
	C21	C22	C23	C24	C25	C26	C27	C28	C29	C30	C31	C32	C33	C34	C35										
**Soil-Mount Fox**																									
MF-01-a-1	15.67	4.02	44.51	8.58	48.40	8.28	109.76	11.14	276.59	30.31	652.26	36.24	255.82	8.50	102.99	3.80	-26.15	1,613	42.45	14.21	1163	4			
MF-01-a-2	5.21	1.68	21.16	3.77	20.76	4.08	53.84	5.00	106.89	12.86	307.15	18.10	134.32	2.17	56.29	8.11	-26.69	753	9.29	14.49	1163	16	0.9978	0.0046	SUERC-85351
MF-01-a-3	5.28	1.84	20.69	3.58	21.69	3.81	62.87	6.14	126.21	14.76	347.12	19.43	131.11	1.87	47.52	7.11	-26.62	814	11.45	14.70	1163	21.5	0.9758	0.0045	SUERC-85352
MF-01-a-4	0.42	0.25	1.22	0.53	2.25	0.67	4.84	0.86	9.28	1.12	24.42	1.33	9.29	0.17	3.14	2.08	-26.24	60	2.88	11.57	1163	26.5	0.8223	0.0038	SUERC-85353
MF-01-a-5	0.24	0.16	0.50	0.34	1.29	0.52	2.99	0.50	5.06	0.50	8.60	0.39	2.95	0.09	0.81	1.89	-25.76	25	1.32	9.88	1163	30.5			
MF-01-a-7	0.06	0.04	0.09	0.08	0.24	0.11	0.68	0.13	1.69	0.11	1.55	0.08	0.40	0.02	0.17	1.53	-24.86	5	0.36	9.34	1163	42.5	0.6642	0.0031	SUERC-85354
MF-01-a-9	0.04	0.02	0.04	0.03	0.08	0.04	0.13	0.04	0.24	0.04	0.51	0.03	0.17	0.01	0.12	0.99	-22.89	2	0.15	7.26	1163	72.5	0.2856	0.0015	SUERC-85355
MF-02-a-2	4.66	1.83	8.93	3.67	33.62	3.80	47.24	3.98	103.08	4.57	129.16	2.65	25.79	3.71	4.68	10.35	-27.59	381	3.68	18.13	1046	23.5			
MF-03-a-2	11.94	8.43	39.65	16.94	158.62	14.87	387.83	20.09	396.60	19.70	206.30	4.35	26.97	20.84	5.29	20.04	-27.84	1,338	6.68	15.11	952	22			
MF-04-a-1	2.39	1.91	8.00	4.38	25.11	5.55	103.82	11.40	317.15	10.69	119.68	3.23	10.95	4.88	0.86	32.68	-28.94	630	1.93	16.25	856	6	1.0898	0.0047	SUERC-85356
MF-04-a-2	1.94	1.66	6.85	3.51	24.46	4.31	75.36	8.96	194.42	8.93	76.64	2.03	7.03	4.79	0.97	24.45	-28.31	422	1.73	13.32	856	14	1.0959	0.0055	SUERC-85357
MF-04-a-3	1.15	1.41	6.49	4.04	26.58	4.82	59.71	7.01	132.79	6.22	56.96	1.73	5.86	5.28	1.21	15.96	-27.68	321	2.01	11.54	856	24			
MF-04-a-6	0.43	0.51	2.57	1.45	11.51	1.69	22.30	2.31	37.68	1.56	16.87	0.43	1.58	0.70	0.35	2.91	-27.60	102	3.50	12.77	856	47.5	0.9203	0.0042	SUERC-85361
MF-04-a-7	0.10	0.14	0.50	0.32	1.42	0.38	2.93	0.51	5.41	0.29	3.07	0.11	0.45	0.08	0.12	1.52	-27.38	16	1.04	8.94	856	51.5	0.8543	0.0039	SUERC-85362
MF-05-a-2	1.31	1.57	6.08	7.92	17.64	7.48	77.16	10.65	191.19	12.23	115.07	3.32	13.61	7.37	1.10	34.35	-28.90	474	1.38	10.03	745	17			
MF-06-a-2	0.77	0.78	4.57	3.10	17.60	3.10	58.84	5.52	110.48	7.24	78.40	3.21	11.72	3.06	0.38	13.04	-28.29	309	2.37	12.51	596	23			
MF-07-b-1	0.67	0.42	1.65	0.74	3.29	1.07	10.89	3.91	71.45	5.44	52.30	2.81	9.10	7.02	0.34	14.32	-29.36	171	1.20	8.89	490	7			
MF-08-a-2	1.47	0.80	3.63	1.08	6.62	1.29	19.48	5.52	94.19	6.17	71.07	2.82	10.58	13.35	0.31	35.84	-28.45	238	0.67	9.44	411	12			
MF-09-a-2	0.76	0.63	2.49	1.20	4.42	1.41	13.38	4.09	68.30	5.82	69.12	3.38	13.71	5.26	0.33			194		9.54	254	16			
**Soil-Alex knob**																									
5.1.1a																21.19	-26.14				1303	6.5			
5.1.1b -1																5.81	-25.93				1303	16			
5.1.1b -2																3.45	-26.17				1303	32			
5.1.1c																0.63	-24.95				1303	64			
5.2.1a																12.81	-28.53				1150	6.5			
5.2.1b																3.13	-27.21				1150	26			
5.2.1c																0.70	-25.94				1150	32			
5.3.1b																7.71	-29.77				1000	8			
5.3.1c																3.35	-29.81				1000	16			

5.3.1d																1.27	-27.44				1000	59			
5.4.1a																11.98	-28.24				905	2			
5.4.1b																7.89	-27.78				905	13			
5.4.1c																3.32	-26.91				905	25			
5.4.1d																1.03	-26.63				905	65			
5.5.1b																2.03	-28.27				694	10.5			
5.5.1c																2.66	-27.72				694	36			
5.5.1d																0.44	-25.64				694	80			
5.5.1e																1.08	-25.90				694	102			
5.6.1a																3.64	-28.47				541	3			
5.6.1b																2.76	-26.82				541	27			
5.6.1c																2.60	-26.86				541	30			
5.8.1b																7.23	-28.01				298	6.5			
5.8.1c																2.35	-26.86				298	30			
**Sediment**																									
PA6m1_S1_37.2	0.27	0.19	0.47	0.23	1.11	0.27	2.50	0.49	6.69	0.52	4.39	0.22	0.77	0.58	0.06	2.46	-28.44	19	0.76	8.20					
PA6m1_S1_54.6	2.56	2.83	3.52	1.77	3.89	0.83	7.83	1.11	15.43	0.87	9.62	0.49	1.83	1.31	0.13	3.63	-28.90	54	1.49	7.97					
PA6m1_S1_74.0	1.35	1.60	2.13	1.02	2.15	0.56	4.89	0.66	9.61	0.55	6.30	0.31	1.29	0.66	0.14	2.86	-28.68	33	1.16	8.31					
PA6m1_S1_104.5	3.62	3.48	2.80	1.36	2.04	0.60	4.24	0.74	8.57	0.60	5.84	0.29	1.22	0.44	0.11	2.07	-28.02	36	1.74	7.16					
PA6m1_S1_111.5	0.58	0.54	0.56	0.48	1.47	0.42	3.47	0.47	4.76	0.38	3.63	0.21	0.93	0.22	0.12	1.54	-27.24	18	1.18	7.84					
PA6m1_S1_118.5	4.01	6.30	6.50	3.60	5.52	1.32	9.58	0.89	10.21	0.73	7.10	0.42	1.96	0.34	0.27	2.55	-27.03	59	2.30	7.10					
PA6m1_S1_120.0	0.80	0.58	1.01	0.69	2.90	0.65	6.90	0.72	8.48	0.47	6.49	0.29	1.68	0.76	0.25	4.28	-27.72	33	0.76	9.22					
PA6m1_S1_124.0	0.95	0.61	0.84	0.52	3.24	0.57	6.13	0.61	7.74	0.40	5.08	0.27	1.16	0.23	0.11	2.47	-26.59	28	1.15	10.52					
PA6m1_S1_126.5	2.87	3.91	4.00	2.22	4.19	0.82	6.33	0.55	6.85	0.38	4.90	0.23	1.23	0.20	0.18	2.18	-27.07	39	1.78	8.19					
PA6m1_S1_130.0	0.55	0.45	0.62	0.40	2.23	0.56	5.37	0.64	6.16	0.49	4.41	0.24	1.07	0.18	0.17	2.10	-27.35	24	1.12	8.67					
PA6m1_S1_133.0	0.34	0.20	0.83	0.30	4.00	0.53	8.91	0.70	10.52	0.54	7.24	0.29	1.61	0.16	0.16	2.98	-26.79	36	1.22	14.09					
PA6m1_S1_134.5	0.30	0.14	0.65	0.23	5.78	0.36	5.96	0.46	7.55	0.39	5.15	0.24	1.17	0.14	0.11	3.61	-26.77	29	0.79	15.69					
PA6m1_S1_136.5	2.97	3.68	4.13	1.76	9.78	0.94	12.64	0.92	14.60	0.78	9.48	0.39	2.08	0.14	0.18	5.62	-27.31	64	1.15	12.78					
PA6m1_S1_140	0.49	0.35	1.24	0.46	8.35	0.70	11.04	0.81	12.92	0.68	8.40	0.32	1.94	0.15	0.17	4.66	-27.15	48	1.03	15.22					
PA6m1_S2_0.0	1.97	1.67	2.04	0.90	2.63	0.73	6.50	0.97	13.16	0.97	8.49	0.42	1.67	0.23	0.12	3.22	-28.22	42	1.32	8.94					
PA6m1_S2_13.0	3.31	2.66	2.36	0.87	2.14	0.58	5.92	1.10	13.93	1.06	8.73	0.43	1.65	0.16	0.11	2.88	-28.42	45	1.56	8.89					
PA6m1_S2_35.3	1.92	1.45	1.88	0.69	2.82	0.71	7.94	1.30	23.05	1.52	17.73	0.82	2.98	0.39	0.14	3.91	-28.88	65	1.67	11.17					
PA6m1_S2_55.0	0.68	0.54	0.74	0.33	1.22	0.47	3.76	0.77	9.00	0.73	6.05	0.31	1.19	0.12	0.10	2.30	-28.13	26	1.13	8.43					
PA6m1_S2_60.0	0.82	0.72	0.66	0.38	0.61	0.29	1.41	0.29	3.21	0.25	2.14	0.14	0.48	0.04	0.06	1.33	-27.98	11	0.86	6.82					
PA6m1_S2_61.7	0.83	0.49	0.56	0.32	1.17	0.34	3.42	0.57	7.49	0.52	5.32	0.26	1.10	0.05	0.10	2.59	-28.58	23	0.87	9.98					
PA6m1_S2_74.4	1.00	0.73	1.11	0.49	1.67	0.47	4.47	0.73	9.75	0.71	6.82	0.31	1.43	0.23	0.15	2.94	-28.53	30	1.02	9.34					
PA6m1_S2_85.9	1.68	1.04	1.19	0.39	1.01	0.36	3.04	0.56	7.04	0.48	4.87	0.24	1.00	0.05	0.11	2.66	-28.83	23	0.87	9.18					
PA6m1_S2_88.6	0.33	0.22	0.26	0.13	0.33	0.13	1.11	0.16	1.91	0.15	1.46	0.08	0.35	0.03	0.03	1.26	-26.67	7	0.53	8.66					

PA6m1_S2_93.6	0.47	0.31	0.38	0.19	0.57	0.18	1.72	0.27	2.83	0.22	2.08	0.12	0.51	0.03	0.06	1.49	-27.01	10	0.67	8.61					
PA6m1_S2_98.6	0.31	0.18	0.31	0.16	0.64	0.22	1.99	0.27	2.63	0.20	1.92	0.12	0.51	0.04	0.06	1.44	-27.11	10	0.66	8.53					
PA6m1_S2_103.6	0.39	0.36	0.42	0.26	0.82	0.27	2.42	0.31	2.88	0.21	2.22	0.12	0.65	0.08	0.06	1.47	-27.03	11	0.78	8.47					
PA6m1_S2_107.2	0.83	0.70	0.91	0.60	1.80	0.68	5.22	0.66	4.93	0.45	3.86	0.25	1.24	0.08	0.14	2.02	-27.19	22	1.11	7.26					
PA6m1_S2_108.9	1.14	0.97	1.14	0.65	2.57	0.48	5.39	0.46	4.93	0.32	3.48	0.19	1.02	0.14	0.07	1.74	-26.36	23	1.32	9.64					
PA6m1_S2_110.6	0.95	0.70	1.30	1.06	2.90	1.17	7.11	0.85	6.52	0.42	4.97	0.24	1.34	0.06	0.10	3.09	-27.68	30	0.96	7.22					
PA6m1_S2_114.6	1.18	0.55	1.17	0.53	2.91	0.55	7.65	0.56	7.54	0.46	6.20	0.27	1.45	0.08	0.09	3.81	-27.31	31	0.82	12.18					
PA6m1_S2_124.6	0.94	0.70	1.28	0.67	2.06	0.57	4.69	0.72	9.89	0.82	6.94	0.39	1.50	0.21	0.10	2.86	-28.37	31	1.10	8.62					
PA6m1_S2_143.6	1.27	1.14	0.98	0.49	0.68	0.34	2.15	0.61	7.35	0.50	5.48	0.31	1.12	0.05	0.06	1.40	-27.35	23	1.60	8.35					
PA6m1_S3_5.0	0.67	0.22	0.97	0.33	2.18	0.48	5.15	0.77	11.36	0.84	8.17	0.38	1.63	0.39	0.13	3.32	-29.05	34	1.02	10.06					
PA6m1_S3_15.0	0.41	0.16	0.55	0.20	0.88	0.24	2.29	0.35	5.12	0.39	3.99	0.22	0.94	0.04	0.08	2.48	-28.52	16	0.64	10.00					
PA6m1_S3_20.5	0.80	0.44	0.75	0.27	0.85	0.25	2.34	0.37	5.37	0.41	4.07	0.21	0.91	0.05	0.08	2.11	-29.11	17	0.81	9.67					
PA6m1_S3_34.5	0.23	0.23	0.26	0.18	0.26	0.15	0.54	0.16	2.48	0.17	1.30	0.10	0.31	0.10		0.60	-28.51	6	1.08	6.73					
PA6m1_S3_56.7	0.95	0.48	0.52	0.24	0.61	0.21	1.70	0.24	2.65	0.21	2.12	0.11	0.56	0.03	0.06	2.44	-27.26	11	0.44	8.47					
PA6m1_S3 60.6	1.52	1.41	1.51	0.90	3.88	0.64	7.47	0.34	6.55	0.61	5.63	0.30	2.01	0.51		2.75	-27.01	33	1.21	9.86					
PA6m1_S3_66.3	0.47	0.30	0.44	0.23	0.74	0.25	2.04	0.27	3.02	0.22	2.45	0.13	0.66	0.04	0.06	1.57	-27.18	11	0.72	8.93					
PA6m1_S3_71.2	0.96	0.60	0.79	0.36	1.27	0.35	3.10	0.35	4.33	0.31	3.54	0.19	0.99	0.04	0.09	1.93	-28.05	17	0.90	9.60					
PA6m1_S3_75.3	0.36	0.28	0.37	0.19	0.64	0.21	1.78	0.27	4.02	0.28	2.72	0.17	0.75	0.27		1.52	-27.64	12	0.81	8.57					
PA6m1_S3_79.2	0.75	0.61	0.66	0.35	1.05	0.33	2.81	0.30	3.36	0.24	2.65	0.15	0.80	0.04	0.08	1.55	-27.35	14	0.91	9.01					
PA6m1_S3_87.1	0.99	0.78	0.93	0.51	1.83	0.53	4.58	0.43	4.10	0.30	3.33	0.18	0.96	0.05	0.10	2.27	-27.12	20	0.86	8.76					
PA6m1_S3_95.7	0.40	0.26	0.41	0.21	0.71	0.25	2.57	0.46	7.45	0.55	9.18	0.32	1.29	0.31		2.36	-28.60	24	1.03	11.58					
PA6m1_S3 111.8	1.65	1.02	1.46	0.64	1.90	0.54	5.58	1.07	12.58	1.02	8.89	0.52	1.75	1.21		1.98	-28.91	40	2.01	7.56					
PA6m1_S3 121.7	1.69	0.99	1.55	0.50	1.84	0.56	4.45	0.96	9.84	0.93	7.13	0.56	1.69	1.41		2.42	-28.98	34	1.41	6.38					
PA6m1_S3 136.7	2.01	1.34	1.65	0.73	2.15	0.62	5.35	1.01	11.08	1.04	8.02	0.55	1.64	1.51		2.60	-28.76	39	1.49	6.56					
PA6m1_S4 0.0	0.72	0.60	0.94	0.53	1.60	0.48	4.14	0.59	6.26	0.51	4.88	0.36	1.28	0.92		1.98	-28.21	24	1.20	6.85					
PA6m1_S4 4.5	1.07	0.99	1.55	0.92	6.00	0.80	8.60	0.88	9.51	0.79	6.58	0.41	1.83	0.89		3.33	-27.24	41	1.23	8.61					
PA6m1_S4 7.9	0.82	0.91	1.28	0.71	2.65	0.54	5.19	0.63	5.83	0.45	4.23	0.28	1.31	0.48		1.97	-26.61	25	1.29	7.73					
PA6m1_S4 16.3	0.86	1.31	1.52	0.93	1.44	0.40	3.10	0.34	3.69	0.29	2.86	0.17	0.85	0.18		1.32	-26.32	18	1.36	7.14					
PA6m1_S4 25.8	0.72	0.66	1.26	0.58	3.62	0.65	8.83	0.62	8.48	0.53	5.69	0.30	1.60	0.34		2.83	-26.98	34	1.20	11.11					
PA6m1_S4 31.7	0.50	0.41	0.70	0.35	1.08	0.28	2.70	0.59	7.04	0.60	5.93	0.37	1.68	0.40		2.96	-28.62	23	0.76	8.34					
PA6m1_S4 37.3	1.44	1.38	1.79	0.93	2.30	0.70	5.87	1.18	17.20	1.67	15.79	0.85	4.07	0.84		3.50	-28.26	56	1.60	8.56					

Table S2. Details of PA6m1 core samples.

Sample Name	Liner Depth (cm)	$\delta^{13}C$ (‰)*	$\delta^{15}N$ (‰)	OC (%)*	C/N*	$F^{14}C$*	Σalk (ug/g sediment)	Λalk (mg/g OC)	$CPI_{n\text{-}alkanes\text{-}}C_{25}\text{-}C_{33}$	Modelled Elevation (m)	-16th percentile	+84th percentile	Modelled Depth (cm)	-16th percentile	+84th percentile
PA6m1_S1_29.7	29.7	-29.10	0.73	4.19	12.80										
PA6m1_S1_32.4	32.4	-28.06	1.91	1.11	8.54										
PA6m1_S1_33.6	33.6	-29.00	0.82	4.26	12.44										
PA6m1_S1_37.2	37.2	-28.44	1.12	2.46	11.63		18.71	0.76	8.20	458	217	242	39	16	18
PA6m1_S1_42.2	42.2	-29.15	1.26	2.58	11.26										
PA6m1_S1_44.6	44.6	-29.03	1.00	3.62	12.10										
PA6m1_S1_46.4	46.4	-28.55	1.39	2.34	11.58										
PA6m1_S1_50.8	50.8	-29.04	0.92	4.05	12.57										
PA6m1_S1_53.3	53.3	-27.72	2.47	2.11	9.86	0.90									
PA6m1_S1_54.6	54.6	-28.90	1.08	3.63	11.98		53.91	1.48	7.97	373	194	225	36	16	17
PA6m1_S1_57.9	57.9	-28.96	0.65	4.80	13.11	0.94									
PA6m1_S1_60.6	60.6	-28.86	0.88	3.26	12.00										
PA6m1_S1_63.1	63.1	-28.98	0.82	4.31	12.94										
PA6m1_S1_68.5	68.5	-28.70	1.20	2.00	10.68										
PA6m1_S1_69.4	69.4	-28.86	0.91	3.42	12.41										
PA6m1_S1_74.0	74.0	-28.68	1.12	2.86	12.01	0.93	33.07	1.15	8.31	428	205	233	36	15	19
PA6m1_S1_79.0	79.0	-28.95	1.25	2.74	11.40										
PA6m1_S1_84.0	84.0	-28.92	1.23	2.79	11.58										
PA6m1_S1_89.0	89.0	-28.69	1.18	2.92	12.19	0.93									
PA6m1_S1_94.0	94.0	-28.23	1.35	2.60	12.92										
PA6m1_S1_99.1	99.1	-27.58	1.48	1.47	10.39										
PA6m1_S1_100.5	100.5	-28.50	1.25	2.30	12.24	0.92									
PA6m1_S1_101.5	101.5	-28.10	1.26	1.87	11.99										
PA6m1_S1_102.5	102.5	-27.74	1.58	1.64	11.40										
PA6m1_S1_103.5	103.5	-28.06	1.88	2.19	10.83										
PA6m1_S1_104.5	104.5	-28.02	1.50	2.07	12.37		35.85	1.73	7.16	460	225	253	48	16	20
PA6m1_S1_105.5	105.5	-28.80	1.34	2.43	10.72	0.92									
PA6m1_S1_106.5	106.5	-28.42	1.74	2.32	11.40										
PA6m1_S1_107.5	107.5	-28.05	1.46	1.99	11.90										
PA6m1_S1_108.5	108.5	-27.68	1.72	1.86	12.01										
PA6m1_S1_109.5	109.5	-28.20	1.93	1.69	11.12	0.90									
PA6m1_S1_110.5	110.5	-27.28	1.72	1.62	11.91										
PA6m1_S1_111.5	111.5	-27.24	1.67	1.54	12.32		18.12	1.18	7.84	626	258	279	51	18	22

PA6m1_S1_112.5	112.5	-27.01	1.51	2.05	14.54	0.91									
PA6m1_S1_113.5	113.5	-27.16	2.02	1.83	11.13	0.89									
PA6m1_S1_114.5	114.5	-26.88	1.35	1.93	14.63										
PA6m1_S1_115.5	115.5	-27.19	1.95	2.25	11.87										
PA6m1_S1_116.5	116.5	-26.89	1.94	2.42	15.06										
PA6m1_S1_117.5	117.5	-27.03	2.01	2.35	13.61										
PA6m1_S1_118.5	118.5	-27.03	1.70	2.55	16.22		58.49	2.29	7.10	609	259	291	58	19	23
PA6m1_S1_119.5	119.5	-27.67	1.84	5.47	15.62										
PA6m1_S1_120.0	120.0	-27.72	1.94	4.28	14.83	0.91	32.43	0.76	9.22	629	237	272	39	17	20
PA6m1_S1_121.0	121.0	-27.55	1.67	3.22	14.81										
PA6m1_S1_121.5	121.5	-27.36	1.71	2.45	15.20										
PA6m1_S1_122.0	122.0	-27.41	1.51	2.53	16.77										
PA6m1_S1_122.5	122.5	-27.26	1.47	2.01	18.02										
PA6m1_S1_123.0	123.0	-26.72	1.53	1.86	19.53										
PA6m1_S1_123.5	123.5	-27.17	1.06	3.82	20.22										
PA6m1_S1_124.0	124.0	-26.59	0.89	2.47	20.36	0.93	28.34	1.15	10.52	887	251	272	42	19	22
PA6m1_S1_124.5	124.5	-25.55	0.95	1.68	25.88										
PA6m1_S1_125.0	125.0	-26.19	0.80	2.99	22.78	0.93									
PA6m1_S1_125.5	125.5	-27.07	1.09	2.36	19.97										
PA6m1_S1_126.0	126.0	-27.07	1.23	1.96	20.11										
PA6m1_S1_126.5	126.5	-27.07	0.80	2.18	20.63		38.67	1.78	8.19	663	252	285	51	19	22
PA6m1_S1_127.0	127.0	-27.18	0.57	3.25	20.93										
PA6m1_S1_127.5	127.5	-27.39	0.49	4.69	22.91										
PA6m1_S1_128.0	128.0	-26.91	0.55	3.14	20.47	0.94									
PA6m1_S1_128.5	128.5	-26.32	0.25	2.58	23.72										
PA6m1_S1_129.0	129.0	-26.68	0.25	3.98	24.92	0.94									
PA6m1_S1_129.5	129.5	-26.62	0.64	3.15	23.13										
PA6m1_S1_130.0	130.0	-27.35	0.91	2.10	20.11		23.37	1.11	8.67	654	246	271	45	18	21
PA6m1_S1_130.5	130.5	-27.20	1.34	1.82	19.54	0.91									
PA6m1_S1_131.0	131.0	-27.05	1.17	1.97	21.37										
PA6m1_S1_131.5	131.5	-27.12	0.98	1.92	19.61	0.92									
PA6m1_S1_132.0	132.0	-26.81	1.06	1.93	23.62										
PA6m1_S1_132.5	132.5	-26.86	0.41	2.33	24.92										
PA6m1_S1_133.0	133.0	-26.79	0.24	2.98	24.90		36.17	1.21	14.09	1041	227	246	23	15	20
PA6m1_S1_133.5	133.5	-26.35	-0.04	3.42	27.55										
PA6m1_S1_134.0	134.0	-26.94	-0.44	4.41	29.37										
PA6m1_S1_134.5	134.5	-26.77	-0.39	3.61	40.85	0.95	28.51	0.79	15.69	1118	222	225	19	13	18
PA6m1_S1_135.0	135.0	-26.62	-0.47	3.19	28.77										
PA6m1_S1_135.5	135.5	-26.42	-0.88	3.55	24.44										

PA6m1_S1_136.0	136.0	-26.50	-0.59	2.85	28.26										
PA6m1_S1_136.5	136.5	-27.31	-0.50	5.62	37.98		64.26	1.14	12.78	897	224	262	25	15	20
PA6m1_S1_137.0	137.0	-27.12	-0.22	4.29	23.94										
PA6m1_S1_137.5	137.5	-26.84	-0.39	5.62	34.10	0.95									
PA6m1_S1_138.0	138.0	-27.13	-0.33	4.99	28.86										
PA6m1_S1_138.5	138.5	-27.09	-0.28	4.49	28.62										
PA6m1_S1_139.0	139.0	-27.24	-0.50	4.93	25.29										
PA6m1_S1_139.5	139.5	-26.97	-0.81	5.37	29.14										
PA6m1_S1_140.0	140.0	-27.15	-0.45	4.66	26.86	0.95	47.86	1.03	15.22	1044	218	239	18	12	18
PA6m1_S1_140.5	140.5	-27.09	-0.50	5.02	30.96										
PA6m1_S1_141.0	141.0	-27.26	-0.44	4.65	26.71										
PA6m1_S1_141.5	141.5	-27.16	-0.16	4.90	31.41										
PA6m1_S1_142.0	142.0	-26.48	0.05	2.86	24.65										
PA6m1_S1_142.5	142.5	-27.39	1.11	3.64	21.80										
PA6m1_S1_143.0	143.0	-27.54	0.33	6.97	26.20										
PA6m1_S1_143.5	143.5	-27.86	1.63	1.45	11.54	0.90									
PA6m1_S1_143.9	143.9	-28.24	1.03	2.92	12.73										
PA6m1_S2_0.0	149.7	-28.22	1.02	3.22	13.58	0.93	42.37	1.32	8.94	534	225	247	37	16	20
PA6m1_S2_5.0	154.7	-28.28	0.85	3.51	14.39										
PA6m1_S2_10.0	159.7	-28.26	0.69	3.39	13.18	0.93									
PA6m1_S2_13.0	162.7	-28.42	1.16	2.88	13.00		44.90	1.56	8.89	501	216	250	35	16	19
PA6m1_S2_15.0	164.7	-28.45	0.98	3.29	15.56										
PA6m1_S2_17.0	166.7	-28.01	1.04	1.54	11.63	0.91									
PA6m1_S2_18.4	168.1	-27.57	2.53	1.35	9.04										
PA6m1_S2_19.4	169.1	-27.08	2.43	0.61	7.59										
PA6m1_S2_20.5	170.2	-29.03	1.34	3.96	12.16										
PA6m1_S2_25.5	175.2	-29.17	1.20	3.51	11.50										
PA6m1_S2_30.5	180.2	-28.88	1.30	3.11	11.55										
PA6m1_S2_35.3	185.0	-28.88	0.99	3.91	12.80		65.18	1.67	11.17	556	195	233	20	13	16
PA6m1_S2_40.3	190.0	-29.06	1.31	3.22	11.96										
PA6m1_S2_45.3	195.0	-27.86	1.86	1.71	10.93										
PA6m1_S2_49.5	199.2	-28.01	1.73	1.37	10.85										
PA6m1_S2_49.8	199.5	-28.18	1.38	2.37	12.43										
PA6m1_S2_55.0	204.7	-28.13	1.05	2.30	12.80		25.93	1.13	8.43	526	223	256	40	17	19
PA6m1_S2_60.0	209.7	-27.98	1.10	1.33	10.47		11.43	0.86	6.82	447	226	260	51	17	21
PA6m1_S2_61.7	211.4	-28.58	1.41	2.59	11.06		22.44	0.87	9.98	540	212	241	28	15	18
PA6m1_S2_66.7	216.4	-28.48	1.48	2.37	11.18										
PA6m1_S2_71.7	221.4	-28.87	1.02	3.25	11.71										
PA6m1_S2_73.3	223.0	-28.76	1.03	3.63	12.37										

PA6m1_S2_74.4	224.1	-28.53	1.27	2.94	12.02		29.93	1.02	9.34	505	205	242	32	15	18
PA6m1_S2_79.6	229.3	-29.00	1.10	4.39	12.21										
PA6m1_S2_80.9	230.6	-28.66	1.56	2.98	11.65										
PA6m1_S2_85.9	235.6	-28.83	1.50	2.66	11.49		22.95	0.86	9.18	453	202	231	30	15	17
PA6m1_S2_88.6	238.3	-26.67	1.77	1.26	14.02		6.65	0.53	8.66	760	268	293	52	20	23
PA6m1_S2_93.6	243.3	-27.01	1.64	1.49	13.35		9.88	0.66	8.61	704	254	294	49	18	23
PA6m1_S2_98.6	248.3	-27.11	1.97	1.44	12.04		9.49	0.66	8.53	684	252	283	48	19	22
PA6m1_S2_103.6	253.3	-27.03	2.11	1.47	10.66		11.41	0.78	8.47	691	254	285	49	19	23
PA6m1_S2_107.2	256.9	-27.19	2.28	2.02	12.70		22.21	1.10	7.26	597	257	276	55	18	22
PA6m1_S2_108.9	258.6	-26.36	1.00	1.74	17.77		22.87	1.32	9.64	865	261	288	48	19	23
PA6m1_S2_110.6	260.3	-27.68	1.80	3.09	14.94		29.60	0.96	7.22	516	237	267	51	18	20
PA6m1_S2_114.6	264.3	-27.31	0.36	3.81	22.52		31.09	0.82	12.18	867	227	265	27	16	20
PA6m1_S2_119.0	268.7	-27.76	1.57	0.99	7.91										
PA6m1_S2_119.5	269.2	-28.06	1.07	2.55	11.20										
PA6m1_S2_124.6	274.3	-28.37	0.74	2.86	10.28		31.36	1.10	8.62	490	214	253	37	17	19
PA6m1_S2_129.6	279.3	-28.48	1.28	2.15	12.38										
PA6m1_S2_134.6	284.3	-28.34	1.30	1.94	13.85										
PA6m1_S2_136.6	286.3	-28.99	1.57	2.74	10.99										
PA6m1_S2_141.6	291.3	-27.58	2.68	1.03	8.84										
PA6m1_S2_142.6	292.3	-28.13	1.41	1.68	11.94										
PA6m1_S2_143.6	293.3	-27.35	2.51	1.40	8.98		22.48	1.60	8.35	634	241	284	47	18	22
PA6m1_S2_144.9	294.6	-28.23	1.44	3.02	12.04										
PA6m1_S3_0.0	300.0	-28.77	1.37	1.87	8.72										
PA6m1_S3_5.0	305.0	-29.05	1.28	3.32	12.04		33.55	1.01	10.06	467	194	227	24	13	17
PA6m1_S3_10.0	310.0	-28.80	1.25	3.45	12.11										
PA6m1_S3_15.0	315.0	-28.52	1.49	2.48	11.69		15.78	0.64	10.00	547	203	244	28	15	18
PA6m1_S3_19.6	319.6	-28.87	1.09	3.20	11.80										
PA6m1_S3_20.5	320.5	-29.11	1.58	2.11	10.69		17.10	0.81	9.67	433	195	226	25	14	17
PA6m1_S3_25.5	325.5	-28.34	1.50	2.45	13.17										
PA6m1_S3_30.5	330.5	-28.58	2.05	1.19	9.19										
PA6m1_S3_34.5	334.5	-28.51	2.33	0.60	7.02		6.49	1.08	6.73	366	201	247	46	16	19
PA6m1_S3_37.6	337.6	-28.15	1.86	1.56	10.34										
PA6m1_S3_41.2	341.2	-26.84	1.50	1.55	14.77										
PA6m1_S3_41.8	341.8	-27.95	1.92	1.79	10.92										
PA6m1_S3_46.8	346.8	-28.48	1.80	1.63	10.91										
PA6m1_S3_50.2	350.2	-27.86	1.61	1.70	12.53										
PA6m1_S3_51.7	351.7	-27.66	1.74	1.44	11.45										
PA6m1_S3_56.7	356.7	-27.26	1.35	2.44	19.07		10.65	0.44	8.47	654	244	280	47	18	22
PA6m1_S3_60.6	360.6	-27.01	0.36	2.75	21.08		33.29	1.21	9.86	784	244	286	42	19	22

PA6m1_S3_66.3	366.3	-27.18	2.14	1.57	11.50		11.25	0.72	8.93	699	248	280	45	18	22
PA6m1_S3_71.2	371.2	-28.05	1.74	1.93	11.80		17.19	0.89	9.60	601	227	252	34	16	19
PA6m1_S3_75.3	375.3	-27.64	2.09	1.52	11.21		12.29	0.81	8.57	609	238	264	43	18	21
PA6m1_S3_79.2	379.2	-27.35	2.46	1.55	10.72		14.09	0.91	9.01	685	249	274	44	18	21
PA6m1_S3_82.4	382.4	-27.51	0.83	3.39	19.99										
PA6m1_S3_83.5	383.5	-27.09	2.01	1.42	14.50										
PA6m1_S3_87.1	387.1	-27.12	1.97	2.27	16.26		19.50	0.86	8.76	696	249	281	47	19	22
PA6m1_S3_90.3	390.3	-27.73	1.72	1.68	13.79										
PA6m1_S3_90.7	390.7	-28.29	1.00	2.44	13.09										
PA6m1_S3_95.7	395.7	-28.60	1.09	2.36	12.26		24.35	1.03	11.58	629	206	243	20	13	17
PA6m1_S3_100.7	400.7	-28.57	1.23	1.96	11.06										
PA6m1_S3_105.7	405.7	-28.28	1.26	1.87	11.44										
PA6m1_S3_111.2	411.2	-27.08	1.86	0.35	7.14										
PA6m1_S3_111.8	411.8	-28.91	1.61	1.98	10.20		39.83	2.01	7.56	351	188	226	38	15	18
PA6m1_S3_116.7	416.7	-29.21	1.59	2.73	10.81										
PA6m1_S3_121.7	421.7	-28.98	1.58	2.42	10.76		34.10	1.41	6.38	283	176	217	44	15	18
PA6m1_S3_126.7	426.7	-28.98	1.78	1.95	10.18										
PA6m1_S3_131.7	431.7	-28.46	1.64	2.31	11.64										
PA6m1_S3_136.7	436.7	-28.76	1.65	2.60	11.33		38.71	1.49	6.56	326	193	228	45	15	19
PA6m1_S3_141.7	441.7	-28.18	1.98	2.11	11.94										
PA6m1_S3_146.7	446.7	-27.97	1.90	1.83	11.91										
PA6m1_S4_0.0	450.5	-28.21	2.01	1.98	11.65		23.81	1.20	6.85	411	220	250	48	16	20
PA6m1_S4_4.5	455.0	-27.24	0.88	3.33	20.33		40.81	1.23	8.61	670	244	283	47	18	21
PA6m1_S4_7.9	458.4	-26.61	1.36	1.97	18.31		25.29	1.29	7.73	717	270	292	58	20	23
PA6m1_S4_11.3	461.8	-26.33	2.38	1.38	12.80										
PA6m1_S4_16.3	466.8	-26.32	2.56	1.32	12.84		17.94	1.36	7.14	724	277	310	63	20	24
PA6m1_S4_20.4	470.9	-26.95	2.43	1.91	12.85										
PA6m1_S4_21.4	471.9	-27.12	2.07	2.25	16.35										
PA6m1_S4_25.8	476.3	-26.98	0.75	2.83	22.95		33.87	1.20	11.11	860	236	278	35	18	21
PA6m1_S4_30.1	480.6	-27.83	1.82	3.21	14.47										
PA6m1_S4_31.5	482.0	-28.25	1.90	1.75	10.58										
PA6m1_S4_31.7	482.2	-28.62	1.26	2.96	13.31		22.61	0.76	8.34	435	207	239	36	15	18
PA6m1_S4_37.3	487.8	-28.26	1.32	3.50	16.38		56.00	1.60	8.56	504	219	249	38	16	20
PA6m1_S4_42.3	492.8	-28.45	1.45	2.04	11.22										
PA6m1_S4_47.5	498.0	-28.85	1.69	2.06	10.17										
PA6m1_S4_48.8	499.3	-27.73	2.83	1.52	7.98										
PA6m1_S4_49.5	500.0	-27.86	1.57	3.70	13.07										
PA6m1_S4_52.5	503.0	-28.94	1.32	2.89	10.88										
PA6m1_S4_57.5	508.0	-28.76	1.46	2.18	10.31										

*data from Frith et al. (ref 21)
